# Immunization with different *Pf*AMA1 alleles in sequence induces clonal imprint humoral responses that are similar to responses induced by the same alleles as a vaccine cocktail in rabbits

**DOI:** 10.1186/1475-2875-10-40

**Published:** 2011-02-14

**Authors:** Kwadwo A Kusi, Bart W Faber, Marjolein van der Eijk, Alan W Thomas, Clemens HM Kocken, Edmond J Remarque

**Affiliations:** 1 Department of Parasitology, Biomedical Primate Research Centre, Postbox 3306, 2280 GH Rijswijk, The Netherlands; 2 Department of Immunology, Noguchi Memorial Institute for Medical Research, College of Health Sciences, University of Ghana, P.O. Box LG581, Legon, Accra, Ghana

## Abstract

**Background:**

Antibodies to key *Plasmodium falciparum *surface antigens have been shown to be important effectors that mediate clinical immunity to malaria. The cross-strain fraction of anti-malarial antibodies may however be required to achieve

strain-transcending immunity. Such antibody responses against *Plasmodium falciparum *apical membrane antigen 1 (*Pf*AMA1), a vaccine target molecule that is expressed in both liver and blood stages of the parasite, can be elicited through immunization with a mixture of allelic variants of the parasite molecule. Cross-strain antibodies are most likely elicited against epitopes that are shared by the allelic antigens in the vaccine cocktail.

**Methods:**

A standard competition ELISA was used to address whether the antibody response can be further focused on shared epitopes by exclusively boosting these common determinants through immunization of rabbits with different *Pf*AMA1 alleles in sequence. Th*e in vitro *parasite growth inhibition assay was used to further evaluate the functional effects of the broadened antibody response that is characteristic of multi-allele vaccine strategies.

**Results:**

A mixed antigen immunization protocol elicited humoral responses that were functionally similar to those elicited by a sequential immunization protocol (p > 0.05). Sequential exposure to the different *Pf*AMA1 allelic variants induced immunological recall of responses to previous alleles and yielded functional cross-strain antibodies that would be capable of optimal growth inhibition of variant parasites at high enough concentrations.

**Conclusions:**

These findings may have implications for the current understanding of the natural acquisition of clinical immunity to malaria as well as for rational vaccine design.

## Background

Malaria caused by parasites of the *Plasmodium spp*. continues to be a major public health problem with half of the world's population at risk of infection [1]. The greatest risk of disease and fatality in *Plasmodium falciparum*-endemic areas occurs in children under 5 years and in first-time pregnant women. Natural immunity to clinical malaria is believed to develop in an age- and exposure-dependent manner, after repeated infection by a number of (different) parasite strains [2-4]. Even in adults who have had several parasite encounters, acquired clinical immunity is partial and is believed to be dependent on constant or periodic exposure to low-level parasitaemia [3,5].

The natural ability to acquire immunity to malaria, although partial, is a strong indication of the feasibility of developing at least an anti-disease vaccine directed against the blood stages of *Plasmodium*. Antigenic variation in immunogenic parasite targets however provides an immune escape route for parasites. Polymorphism in such well-known vaccine targets as the Merozoite Surface Proteins (MSPs) and Apical Membrane Antigen 1 (AMA1) have been associated with host immune pressure on parasites [6-10]. This presents malaria vaccine researchers with a formidable challenge since immunization with one variant of these polymorphic antigens induces antibodies that show limited cross-inhibition/recognition of parasites expressing other allelic variants of the same antigen. This has been demonstrated extensively in animal models [11,12] and to some extent in human field studies [13-16].

There is growing interest in multi-allele/multi-antigen malaria vaccines and the potential of such vaccines for the induction of broad inhibitory antibody responses has been demonstrated [17-19]. The broadened response most likely results from diluting out strain-specific epitopes in the antigen mixture, with the bulk of remaining epitopes being those that are common to the vaccine component alleles [20].

The hypothesis that immunization of rabbits with different *Pf*AMA1 alleles in sequence would result in boosting of only antibodies to epitopes that are common to all antigens was tested in this study. Antibodies to highly specific epitopes would not be boosted, and this is expected to further increase the proportion of induced cross-strain antibodies in comparison with antibodies induced by a multi-allele vaccine that incorporates the same allelic antigens. Such a mechanism of cross-strain antibody production would be based on the concept of original antigenic sin (clonal imprinting). Original antigenic sin results when prior exposure to one strain/antigen diverts the antibody response to shared epitopes following exposure to a second closely related strain/antigen such that the newly elicited antibodies still react strongly with the priming antigen [21-23]. A sequential immunization protocol may mimic the development of natural clinical immunity and provide some insight into its acquisition in the field where over time an individual is exposed (sequentially) to a number of variant parasite strains. The generated data shows that a sequential immunization protocol may not be materially different from a mixed antigen protocol with respect to the proportions of elicited strain-specific and cross-strain antibodies. As expected, antibody production in the sequential immunization groups was through associative immune recall of previous antigen encounter. This data is relevant to the current understanding of the acquisition of clinical immunity against malaria in endemic areas, as well as for rational vaccine design.

## Methods

### Antigen production, rabbit immunization and antibody purification

The full ectodomain of the AMA1 allelic forms from *P. falciparum *strains FVO, HB3, 3D7 and CAMP, as well as the *in silico*-designed *Di*versity *Co*vering antigens (DiCo 1, DiCo 2 and DiCo 3) [24], were expressed as recombinant proteins in *Pichia pastoris*. The three DiCo proteins were all expressed with the FVO AMA1 prodomain, and all antigens were mutagenized at up to six potential N-glycosylation sites within the *Pf*AMA1 ectodomain. The expression, purification and characterisation of all antigens were as described previously [25].

Rabbit housing and immunization were at BioGenes GmbH (Berlin, Germany), and were in accordance with national and international animal welfare regulations. Rabbit immunization at this facility was under approval from NIH/OLAW (ID number #A5755-01). Five groups of rabbits were immunized intramuscularly with three doses (30 μg per dose) of different *Pf*AMA1 vaccine formulations either in sequence or as an antigen cocktail on days 0, 28 and 56. All vaccines were formulated in a modified Freund's adjuvant (95% paraffin oil, 2.4% Tween 40, 0.1% cholesterol and 0.01% lipo-polysaccharide from blue-green algae) provided by BioGenes, and formulation was according to the manufacturer's protocols. Three of the groups were immunized with *Pf*AMA1 alleles from the FVO, HB3 and 3D7 strains of *P. falciparum *in different orders (details in Table 1). A fourth group was immunized with three doses of an equimolar mixture of these three alleles (designated as NA mix, 10 μg of each allele, 30 μg dose), and the last group was immunized with three doses of an equimolar mixture of the three DiCo proteins (10 μg of each DiCo, 30 μg dose), referred to as DiCo mix (Table 1). Rabbits were exsanguinated on day 70 and sera from all five groups were analysed in ELISA while purified antibodies from these sera were used in growth inhibition assays described here.

**Table 1:** Schedule indicating the order of *Pf*AMA1 antigen administration to rabbits.

Immunization group	Antigens and immunization			*Number of rabbits
		
	Day 0	Day 28	Day 56	
1 (f3h)	FVO AMA1	3D7 AMA1	HB3 AMA1	6
2 (hf3)	HB3 AMA1	FVO AMA1	3D7 AMA1	6
3 (h3f)	HB3 AMA1	3D7 AMA1	FVO AMA1	4
4 (NA mix)	NA mix	NA mix	NA mix	6
5 (DiCo mix)	DiCo mix	DiCo mix	DiCo mix	6

***f ***(FVO), ***3 ***(3D7) and ***h ***(HB3) in groups 1, 2 and 3 indicate the order of antigen administration.

NA mix - an equimolar mixture of AMA1 antigens from the FVO, HB3 and 3D7 strains of *P. falciparum*, administered at all three immunization time points.

DiCo mix - an equimolar mixture of the three Diversity-covering (DiCo) proteins, administered at all three immunization time points.

Antibodies from final bleed sera were purified on Protein G Sepharose (GE Healthcare, Etten-Leur, The Netherlands) columns. Binding and elution buffers (Pierce, Rockford, IL) were used according to manufacturer's protocols. After elution, antibody eluates were filtered (0.22 μm), concentrated and exchanged into RPMI 1640 using pre-sterilized Amicon Ultra-15 tubes (30-kDa cutoff; Millipore, Ireland). The concentration of each antibody fraction was subsequently determined by a Nanodrop ND1000 spectrophotometer (Nanodrop Technologies, Wilmington, DE) using the IgG extinction coefficient, adjusted to 12 mg/ml and stored at -20°C until use.

### ELISA and growth inhibition assays

Sera from all rabbits were titrated in a standardized ELISA on plates coated with recombinant AMA1 allelic antigens from FVO, HB3 or 3D7 parasite strains. Sera were also analysed with a harmonized competition ELISA protocol that has been described elsewhere [20]. FVO, HB3 and 3D7 AMA1 proteins were used as capture antigens and FVO, HB3, 3D7 and CAMP AMA1 antigens were used as competitor antigens in all assays.

Protein G-purified IgG fractions from final bleed sera were tested for *in vitro *activity in parasite growth inhibition assays (GIAs). All IgGs were tested in triplicate on FCR3 (one amino acid difference in the pro-domain from the FVO strain, with *ama1 *GenBank accession no. M34553), NF54 (parent strain of the 3D7 clone with *ama1 *GenBank accession no. U65407), HB3 (accession no. U33277), 7G8 (accession no. M34555) and CAMP (accession no. M34552) parasite strains at a 2-fold serial dilution from 6 mg/ml in 96-well half area cell culture plates (Greiner, Alphen a/d Rijn, The Netherlands). Parasites were cultured under standard conditions (an atmosphere of 5% CO_2_, 5% O_2_, and 90% N_2_, 37°C), and all parasite strains were verified by PCR and restriction fragment length analysis of the *Pf*AMA1 antigen they express. Parasite cultures were mycoplasma-free and synchronized with 0.3 M Alanine, 10 mM Hepes, pH 7.5 before use in assays. Late trophozoite/early schizont stages at a parasitaemia of 0.3 ± 0.1% and 2% final haematocrit were used in all assays. The final culture volume was 50 μl/well and parasites were incubated for 42-46 h. Parasite growth was assessed by measuring parasite lactate dehydrogenase levels and plates were read at 655 nm after 30 min of development. Parasite growth inhibition was expressed as 100 - ((*A_655_Sample - A_655_RBC)/(A_655_SZ - A_655_RBC)) × 100*, where *A_655_Sample *is the OD_655 _for any test sample well, *A_655_SZ *is the average OD_655 _of schizont control wells included on each plate and *A_655_RBC *is the average OD_655 _of RBC control wells. The data is presented as the arithmetic mean% inhibition from each sample triplicate.

### Statistical analyses

All analyses and graphics were made using the *R *statistical package (R Development Core Team, 2009, version 2.10.1). ELISA antibody titres in day 70 sera were log-transformed and compared between groups by one-way analysis of variance (ANOVA) followed by the pair-wise Tukey Honest Significant Difference post hoc test which applies a correction for multiple comparisons. Titres are also presented as dotplots superposed with boxplots indicating the median, lower and upper quartiles. Residual antibody binding (***Y_min_***) for each competitor antigen in competition ELISA was estimated by a 4-parameter logistic fit with least squares approximation. The mean % depletion (100-***Y_min_***) and corresponding 95% confidence intervals (95% CI) for each competitor antigen are presented for all immunization groups. GIA data is presented as the mean % growth inhibition ± standard error of mean per immunization group against the five parasite strains. Associations between antibody titre and the corresponding *in vitro *parasite growth inhibition levels were estimated with a four-parameter logistic fit. Differences were considered statistically significant at p < 0.05, or when the 95% CI of groups being compared did not overlap.

One of the 6 rabbits in the first immunization group (f3h) experienced pneumonia during the study and data from this rabbit was excluded from all analyses. Rabbits (n = 4) in one of the sequential groups (h3f, Table 1) were immunized in a different experiment.

## Results

### Specificity of antibodies elicited with mixed allele and sequential allele immunization protocols

**Figure 1:**
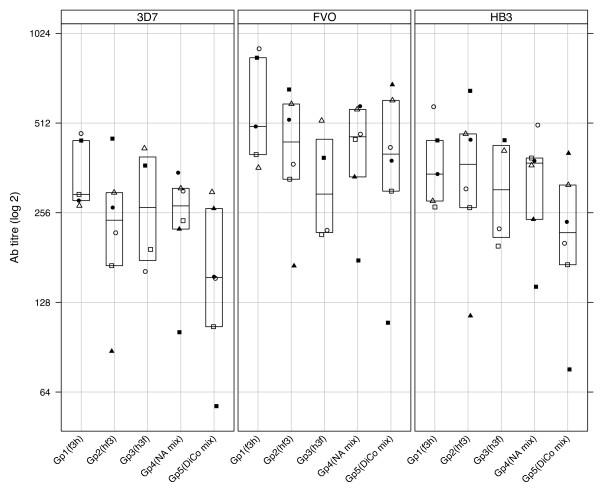
**Absolute levels of anti-AMA1 antibodies elicited with mixed allele and sequential allele protocols in rabbits**. Rabbits in groups 1 - 3 were immunized with three *Pf*AMA1 allelic antigens (from FVO, HB3 and 3D7 parasite strains) in three different sequences (refer to Table 1). The fourth group of rabbits was immunized with a cocktail of the three *Pf*AMA1 alleles, while the fifth group was immunized with a mixture of the three Diversity covering proteins (DiCo mix) at all immunization time points. All vaccines were formulated with a modified Freund's adjuvant containing a lipo-polysaccharide from blue-green algae as adjuvant. Antibody titres of sera taken on day 70 were determined by a standardized ELISA with 3D7 (left panel), FVO (middle panel) and HB3 (right panel) AMA1-coated plates. Data is presented on a Log2 scale as dotplots with a boxplot superpose indicating the median, lower and upper quartiles per immunization group. For each capture antigen, plotting symbols represent the antibody titre of individual rabbits within an immunization group.

Antibodies from rabbit sera drawn on day 70 from all five groups were titrated against *Pf*AMA1 alleles from FVO, HB3 and 3D7 parasite strains, and the data is presented in Figure 1. There were no statistically significant differences for comparisons of the log-transformed antibody titres either for any single immunization group against all three capture antigens, or for the different immunization groups against the same capture antigen (P > 0.05, one-way ANOVA). Pair-wise comparison of antibody titres for an immunization group against any two capture antigens, or titres for any two groups against the same capture antigen also showed no significant differences (P > 0.05, Tukey HSD).

**Figure 2:**
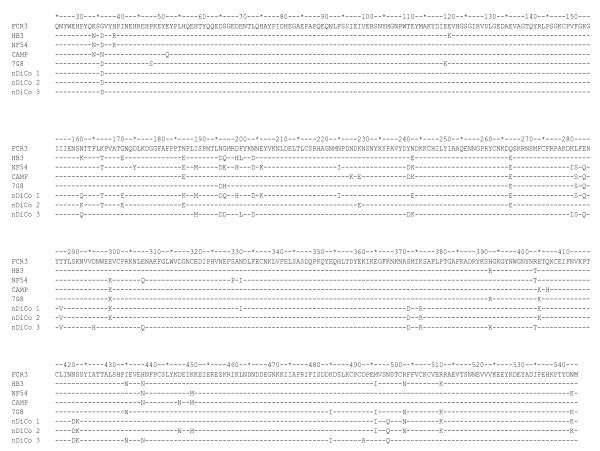
**Protein sequence (aa 25 - 545) alignments for DiCo antigens and parasite AMA1 alleles**. The recombinant AMA1 allelic antigens for FVO, HB3, 3D7 and CAMP used in ELISA differ at 6 positions (5 for HB3 AMA1) from the respective parasite sequences presented here. These differences (N162Q, T288V, S373 D, N422 D, S423K, N499Q) were introduced in the recombinant antigens to prevent N-glycosylation of the *Pichia pastoris*-expressed antigens.

Competition ELISA was performed to assess the relative proportions of cross-reactive and strain-specific antibodies induced against the individual vaccine antigens and a fourth *Pf*AMA1 allele (CAMP) that was not a component of any of the vaccines. The FVO, HB3 and 3D7 AMA1 allelic proteins were used as capture antigens and FVO, HB3, 3D7 and CAMP AMA1 as competitor antigens. Depletion (%) of antibodies with the different competitor antigens against each capture antigen is presented in Table 2. On each capture antigen, complete depletion of antibodies by the homologous competitor antigen was observed as expected. Heterologous antibody depletion was however dependent on the number of amino acid differences between the capture and competitor antigens (presented in Figure 2). For example, depletion was lowest for 3D7 AMA1 competitor antigen when FVO AMA1 was used as capture antigen, and lowest for CAMP AMA1 when HB3 AMA1 was used as capture antigen (Table 2).

**Table 2:** Mean % antibody depletion from FVO, HB3 and 3D7 AMA1-coated plates.

Coating antigen	Competitor antigen	Gp 1 (f3h) n = 5	Gp 2 (hf3) n = 6	Gp 3 (h3f) n = 4	Gp 4 (NA mix) n = 6	Gp5 (DiCo mix) n = 6
FVO AMA1	FVO	98.5 (97.3 - 99.7)	95.9 (95.1 - 96.8)	98.2 (96.3 - 100.1)	96.8 (95.4 - 98.2)	97.6 (96.4 - 98.7)
	HB3	93.2 (88.1 - 98.3)	90.4 (87.0 - 93.8)	93.2 (86.0 - 100.4)	90.6 (87.4 - 93.9)	91.3 (89.7 - 92.9)
	3D7	84.5 (77.5 - 91.5)	75.5 (74.3 - 76.7)	76.6 (65.4 - 87.8)	70.4 (64.7 - 76.0)	77.0 (69.4 - 84.7)
	CAMP	78.7 (73.6 - 83.7)	70.3 (65.9 - 74.6)	74.2 (64.4 - 84.1)	70.3 (64.7 - 74.0)	84.0 (82.3 - 85.8)
						
HB3 AMA1	FVO	87.5 (79.0 - 96.0)	86.0 (81.2 - 90.9)	91.4 (89.3 - 93.6)	86.6 (83.3 - 89.9)	84.1 (80.6 - 87.5)
	HB3	96.1 (93.6 - 98.5)	97.5 (95.4 - 99.6)	98.2 (98.1 - 98.4)	96.7 (95.0 - 98.4)	95.6 (93.7 - 97.4)
	3D7	86.7 (82.3 - 91.0)	84.3 (75.9 - 92.8)	77.9 (70.5 - 85.4)	73.7 (66.7 - 80.6)	74.7 (70.5 - 78.9)
	CAMP	76.8 (73.4 - 80.1)	68.5 (62.7 - 74.3)	74.4 (70.2 - 78.6)	66.8 (60.2 - 73.3)	81.2 (78.2 - 84.2)
						
3D7 AMA1	FVO	83.0 (75.3 - 90.8)	91.8 (86.7 - 97.0)	91.3 (84.8 - 97.9)	73.2 (66.1 - 80.3)	86.9 (84.4 - 89.4)
	HB3	89.3 (85.2 - 93.4)	95.9 (92.7 - 99.1)	94.1 (89.2 - 99.0)	83.6 (76.1 - 91.1)	88.7 (86.2 - 91.2)
	3D7	100.2 (96.8 - 103.7)	98.2 (95.9 - 100.2)	96.8 (94.6 - 99.0)	95.8 (94.3 - 97.4)	97.1 (95.2 - 99.0)
	CAMP	79.5 (71.6 - 87.5)	82.0 (77.0 - 87.1)	85.7 (77.0 - 94.5)	71.0 (66.5 - 75.5)	89.2 (86.0 - 92.4)

Values reported as mean (95% CI) per immunization group for the same competitor antigen on each coating antigen.

Comparison of the three sequential immunization groups (f3h, hf3 and h3f) showed that though there were small differences (based on overlaps in 95% CI, Table 2) in the extent of heterologous antibody depletion, no clear trends emerged with respect to the order of antigen administration and the capture antigen used in assays. The three sequential immunization groups also showed detectable quantities of antibodies that were specific to each of the three vaccine *Pf*AMA1 alleles in all polyclonal pools on day 70, despite the fact that each allelic antigen was administered at only one of the three time points.

Comparisons across all groups showed that antibody depletion by heterologous competitor antigens was generally lowest in the NA mix immunization group compared to the three sequential immunization groups (f3h, hf3 and h3f, Table 2). The observed differences were however not always statistically significant since 95% CI sometimes overlapped. Antibody depletion by CAMP AMA1, an allele that was not in any of the vaccine formulations, was greatest for antibodies from the DiCo mix vaccine group compared to the other four groups in all assays (Table 2). CAMP AMA1 depletion of anti-DiCo mix antibodies was statistically significantly higher than that of anti-NA mix antibodies irrespective of the capture antigen, while differences between anti-DiCo mix antibodies and the sequential immunization groups were not always statistically significant (Table 2).

### Functional capacity of antibodies elicited with mixed allele and sequential allele immunization protocols

**Figure 3:**
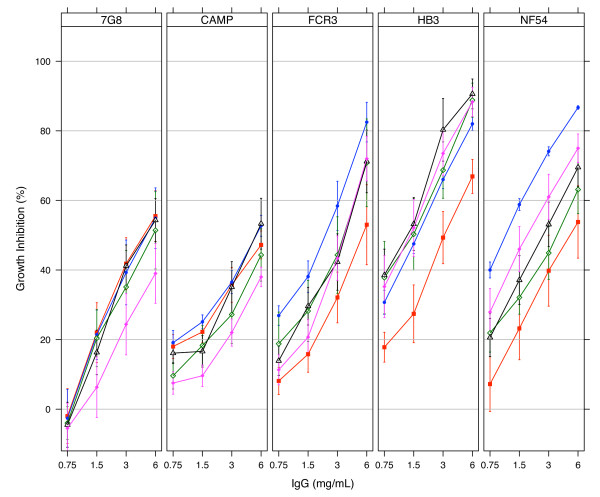
**Growth inhibition of *P. falciparum *parasites by antibodies elicited with mixed and sequential allele protocols**. Protein G-purified antibodies from all immunization groups were tested on each of five culture-adapted strains (7G8, CAMP, FCR3, HB3 and NF54) of *P. falciparum*. Plots represent the mean % inhibition ± SEM for all antibody samples within an immunization group. Data for group 1 (f3h, n = 5) is plotted in blue, group 2 (hf3, n = 6) in black, group 3 (h3f, n = 4) in green, group 4 (NA mix, n = 6) in magenta and group 5 (DiCo mix, n = 6) in red.

Protein G-purified antibodies from day 70 bleeds were used for *in vitro *growth inhibition assays. Antibodies from the three sequential immunization groups (f3h, hf3 and h3f) at 6 mg/ml showed similar mean levels of inhibition of two of the three parasites expressing the vaccine alleles (FCR3, p = 0.60; HB3, p = 0.28; one-way ANOVA) irrespective of the order of antigen administration (Figure 3). Mean inhibition levels against the NF54 strain were however higher for antibodies from the f3h group compared to those from the h3f group (p = 0.02, Tukey HSD). Pair-wise comparisons of mean growth inhibition levels in any sequential immunization group with that of the NA mix group against any of the parasites showed no significant differences (p > 0.05, Tukey HSD) despite the weak trend of high antibody depletions from the sequential immunization groups in competition assays (Table 2). Thus it did not matter whether the vaccine was administered as a mixture or in sequence, the functional outcome *in vitro *was the same.

Antibodies from the sequential (f3h, hf3 and h3f) and NA mix immunization groups generally showed a reduction in the extent of *in vitro *inhibition of "heterologous" parasite strains (CAMP, 7G8) compared to that of "homologous" strains (NF54, FCR3, HB3). Mean inhibition with antibodies from both the f3h (Gp 1) and NA mix (Gp 4) immunizations at 6 mg/ml were all higher against "homologous" strains (NF54, HB3, FCR3) compared to those against the CAMP and 7G8 strains (p < 0.0001, one-way ANOVA). Mean inhibition of antibodies from the hf3 group (Gp 2) were however only higher against HB3 strains when compared pair-wise with the "heterologous" strains (p = 0.006 for 7G8 and p = 0.005 for CAMP parasites, Tukey HSD). Antibodies from the h3f group (Gp 3) also showed higher mean inhibition against HB3 in comparison with the CAMP strain (p = 0.024, Tukey HSD). In contrast, antibodies from the DiCo mix group (Gp 5) showed a generally consistent level of inhibition of all five parasite strains (Figure 3). Mean growth inhibitions ranged from 47.2% against the CAMP strain to 66.9% against the HB3 strain at 6 mg/ml total IgG, and these were not statistically significantly different (p = 0.55, one-way ANOVA).

**Figure 4:**
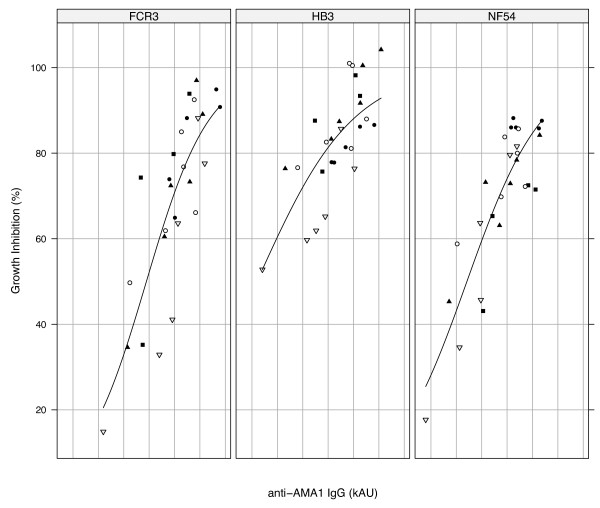
**ELISA antibody titre correlates with *in vitro *parasite growth inhibition**. Association of antibody levels with *in vitro *antibody functionality is shown for parasite strains FCR3 (FVO), HB3 and NF54 (3D7). *In vitro *inhibition of any parasite strain at 6 mg/ml of purified antibody has been plotted against antibody titres measured with the corresponding AMA1 allele. Plots are based on a four-parameter logistic function. Filled circles represent animals in group 1 (f3h, n = 5), filled triangles represent group 2 (hf3, n = 6), filled squares represent group 3 (h3f, n = 4), open circles represent group 4 (Na mix, n = 6) and open triangles represent group 5 (DiCo mix, n = 6).

The data generally suggests that effectiveness of the antibody response was dependent on the test parasite strain ("homologous" vs. "heterologous"), and the absolute levels of elicited antibodies. Higher antibody titres are expected to give greater *in vitro *parasite growth inhibition levels since antibody titres against specific alleles correlate well with the level of *in vitro *inhibition of parasites expressing those alleles (Figure 4).

## Discussion

An effective malaria vaccine is expected to confer similar or better immunity to malaria-susceptible individuals compared to that of adults who are resident in endemic areas, but over a shorter period of time. In semi-immune adults, this level of anti-disease immunity is acquired after repeated infection with diverse parasite strains [2,3]. Antibodies are key components of anti-disease immunity [26,27], and the cross-strain fraction of anti-malarial antibodies may be important effectors against parasite strains that express diverse polymorphic antigens [7,14,28]. The main objectives of this study were to compare sequential and mixed *Pf*AMA1 immunization protocols for the proportions of functional cross-strain antibodies they induce in rabbits, and to further demonstrate the specificity broadening effects of such vaccination strategies.

Statistically similar levels of antibodies were induced with all vaccine formulations, irrespective of the order of antigen administration (Figure 1). This suggests that the order of antigen exposure may not influence the levels of elicited antibodies. For sequential immunization groups, this shows that "booster" responses were associative recall responses of previous allele vaccinations, and antibodies were mostly to epitopes that are shared by vaccine alleles. This is consistent with published data on both cellular and humoral immune responses to other polymorphic malaria antigens [7,29-31], and is a well-established phenomenon in immune responses to other parasitic and viral infections [21,22,32-34].

Antibody depletion data from competition assays showed marginally higher proportions of cross-strain antibodies in some sequential immunization groups compared to the NA mix group (Table 2). Since significance was achieved only in some instances and there were no observable trends with respect to capture and competitor antigens, the order of antigen administration may only marginally influence the ultimate specificity of antibodies on day 70. The three-antigen DiCo mix vaccine generally yielded higher proportions of cross-strain antibodies compared to the three-antigen NA mix vaccine, especially against the out-group competitor antigen CAMP (Table 2). This suggests that the three DiCo antigens together present a greater proportion of epitopes that induce broad-reacting antibodies, and affirms the specificity broadening properties of the DiCo vaccine approach [9,35].

The determination of strain-specific antibodies against vaccine alleles in sequential immunization groups on day 70 (Table 2) suggests that immunization with a single allele does induce long-lived responses. This may reflect the persistence of strain-specific antibodies that were induced after antigen administration on day 0 (first antigen), 28 (second antigen) or 56 (third antigen). Alternatively, memory B cells to these specific antibody epitopes in previous vaccine alleles could be activated by the altered, corresponding low affinity epitopes on subsequently administered *Pf*AMA1 alleles, leading to high affinity secondary responses against the cognate epitopes. The latter phenomenon is in agreement with the mechanism underlying original antigenic sin, and supports the existence of a continuum of antibody specificities [7]. It must be noted that the specificity of an antibody for an antigen is directly related to the affinity of the antigen-antibody interaction hence an antibody that is "specific" to one *Pf*AMA1 allele may indeed have very low affinity for other alleles.

Data from *in vitro *growth inhibition assays was consistent with the observations in ELISA. The similar inhibition of FCR3, HB3 and NF54 parasite strains by purified antibodies from sequential immunization (f3h, hf3 and h3f) and NA mix groups suggests that comparable levels of functional antibodies against all three parasite strains were present on day 70, irrespective of the order of antigen administration (Figure 3). This confirms the induction of antibodies mostly to shared epitopes based on the original antigenic sin phenomenon [7,22].

The observed higher inhibition of parasite strains expressing the vaccine alleles compared to the out-group strains (CAMP, 7G8) may be attributed to the generally low levels of antibodies induced in all immunization groups. A similar observation was made in an earlier study where low titres of antibodies elicited against DiCo mix in Montanide IMS resulted in lower *in vitro *parasite inhibition levels compared with the higher antibody levels against DiCo mix in two other adjuvants [35]. ELISA antibody titres and parasite inhibition levels in the present study are intermediate between those of anti-DiCo mix antibodies elicited in the previous study with Montanide IMS on the one hand and CoVaccine HT™/Montanide ISA 51 on the other hand. Similar proportions of cross-strain antibodies were measured in both studies despite the different absolute antibody titres. At such low antibody titres, high avidity strain-specific antibodies, which form a small proportion of elicited antibodies, most likely augment the functional effects of cross-strain antibodies against the respective homologous parasite strains. This fraction of strain-specific antibodies would however have very low avidities for AMA1 of the CAMP and 7G8 strains, resulting in lower levels of inhibition of these strains (Figure 3). Thus high titres of functional cross-strain antibodies are required to optimally inhibit "heterologous" strains. At high antibody titres, the additional inhibitory activity of any strain-specific antibodies against "homologous" parasites would put the overall inhibitory effect in the upper plateau region of the antibody binding-function curve (Figure 4). A possible limitation here however, is that some parasites, here the HB3 strain, may be inherently easier to inhibit than others, and this could mask the effect of vaccine responses described. Similar effect has been described for D10 parasites, which can be inhibited better with anti-3D7 AMA1 antibodies compared to inhibition of the homologous 3D7 strain [36,37].

Although anti-DiCo mix antibodies least inhibited the three "homologous" strains, these antibodies performed as well against the two out-group parasite strains as antibodies from all other vaccine formulations. Additionally, unlike the other vaccine formulations, anti-DiCo mix antibodies showed consistent inhibition of all parasite strains. This consistency, coupled with the significantly higher depletion of anti-DiCo antibodies by the out-group CAMP AMA1 competitor antigen when compared to anti-NA mix antibodies, suggests that DiCo mix may have a slight advantage as a vaccine candidate, since AMA1 from culture-adapted strains may not necessarily be encountered in the field.

The current *in vitro *growth inhibition data may seem inconsistent with our earlier published data [20], where rabbit antibodies elicited with a three-antigen (FVO, HB3, 3D7 AMA1) vaccine in Montanide ISA720 inhibited a heterologous parasite strain (CAMP) to a similar extent as the three "homologous" strains. This can however be explained by the fact that data from the earlier study was based on a single sample per immunization group, with antibody titres that were 2-4 times higher than the average antibody titres in the study under discussion. The greater potency of the adjuvant used in the earlier study may partially account for the higher titres. Additionally, the earlier study used an immunization protocol (4 vaccine doses on days 0, 28, 56 and 82, exsanguination on day 95) that is different from the one used in the current study (3 doses on days 0, 28 and 56, exsanguination on day 70). The extra booster dose, as well as the longer study period, could account partially for the higher functional antibody titres, which were most likely at saturation levels. It must be noted that single allele immunizations with FVO, HB3 and 3D7 AMA1 alleles in the previous study also resulted in similar antibody titres to that of the three-antigen mixed allele vaccine but failed to achieve the same level of *in vitro *inhibition of heterologous parasites as the mixed allele vaccine [20]. Moreover, mixed allele vaccine antibodies from both studies showed similar binding specificities for the component antigens. Thus high titres of cross-strain antibodies are necessary for significant inhibition of variant parasites, and this is consistent with earlier published literature in both *in vitro *and *in vivo *settings [28,35].

This data may aid current understanding of the acquisition of clinical immunity to malaria in endemic areas. Induction of antibodies to polymorphic antigens in the field upon infection with different parasite strains may be through an original antigenic sin mechanism, and individuals will most likely accumulate a strain-transcending repertoire of antibodies over time. This will also explain why clinical testing of a mono-allelic vaccine based on a polymorphic antigen in an unexposed population yields antibodies that react better with homologous than heterologous antigenic alleles [13] while antibodies taken after a similar trial in a malaria-endemic population react equally well with both homologous and heterologous vaccine alleles [38]. A mixture of strain-specific and cross-strain antibodies are most likely induced in naïve individuals in the former instance while previous exposure in the latter results in a boost of responses to epitopes that are common to the vaccine and previously encountered alleles.

In summary, a mixed antigen immunization protocol is expected to elicit humoral responses similar to those elicited by a sequential immunization protocol, and by extension the response induced naturally in individuals in malaria-endemic populations. Thus the anti-AMA1 component of a natural immune response can be effectively mimicked by immunization with a cocktail of AMA1 alleles. This finding may also apply to the many other polymorphic parasite antigens that are currently undergoing clinical evaluation. Additionally, sequential exposure to different AMA1 alleles induces immunological recall of responses to previous alleles and yields functional cross-strain antibodies that are capable of optimal parasite growth inhibitions at high enough concentrations. These findings may aid current understanding of the natural acquisition of clinical immunity to malaria as well as provide fresh insight into rational vaccine design.

## Competing interests

Four of the authors are in the process of obtaining a patent for the three synthetic Diversity-Covering (DiCo) AMA1 proteins. This does not alter their adherence to any Malaria Journal policies on sharing data and materials.

## Authors' contributions

Conceived and designed the experiments: EJR KAK BWF CHMK AWT. Performed the experiments: KAK MvdE. Analysed the data: KAK EJR BWF. Designed and produced recombinant proteins: BWF, MvdE. Wrote the paper: KAK EJR BWF CHMK AWT. All authors have read and approved the final manuscript.
